# Oral Cancer Awareness and Knowledge in the City of Valongo, Portugal

**DOI:** 10.1155/2012/376838

**Published:** 2012-08-07

**Authors:** Luís Silva Monteiro, Filomena Salazar, Júlio Pacheco, Saman Warnakulasuriya

**Affiliations:** ^1^Medicine and Oral Surgery Department, Dental Sciences Group, Health Sciences Research Centre, Instituto Superior de Ciências da Saúde Norte, Rua Central de Gandra 1317, Paredes, 4585-116 Gandra, Portugal; ^2^Stomatology Department, Centro Hospitalar de São João, Polo de Valongo, 4440-563 Valongo, Portugal; ^3^Oral Medicine, Department of Clinical and Diagnostic Sciences and WHO Collaborating Centre for Oral Cancer, King's College London Dental Institute, London SE5 9RW, UK

## Abstract

We conducted a questionnaire survey among 602 subjects in order to analyze the awareness and knowledge on oral cancer among residents of the city of Valongo in Portugal. The cancer that most subjects were aware of was breast cancer (99%). Oral cancer was the least mentioned cancer (68.6%). There was awareness of the relationship between oral cancer and smoking among 89.5% subjects, but less of the association with alcohol misuse (63.3%). Nonhealing mouth ulcers were identified as a sign or symptom of oral cancer by 90.0% and red or white patch by only 52.8% subjects. Whereas 94.5% agreed that early detection could improve the treatment outcome, a disheartening 28.1% believed that whether a person developed an oral cancer or not is a matter of luck and therefore is unavoidable. Surprisingly only 1.7% were ever submitted to or had knowledge of receiving a consultation regarding oral cancer. In conclusion, this survey demonstrates a general lack of awareness and knowledge on oral cancer in a population of Valongo. An oral health promotion strategy should involve elements of basic education on oral cancer for this population, and regular oral cancer screenings should be implemented in Valongo.

## 1. Introduction

Oral cancer is a major public health problem worldwide [1]. Oral and pharyngeal cancers grouped together are the sixth most common cancers in the world. The most recent estimated world incidence of oral cancer is around 263,861 new cases for the year of 2008. In Portugal in the same year, oral cancer was the 6th most frequent cancer in males, with 777 new cases (age-standardized incidence of 9.9/100,000 habitants) and 248 new cases among females (age-standardized incidence of 2.1/100,000 habitants) [2]. 

Almost 90% of oral cancers are squamous cell carcinomas [3]. Smoking, alcohol use, smokeless tobacco products, and HPV infections are the major risk factors, with an attributable risk of oral cancer due to both tobacco and alcohol of 80% [4].

Despite recent advances in the detection and treatment of cancer, visual accessibility of the oral mucosa, and the scientific knowledge on cancer risk factors, oral cancer carries a low survival rate (near 50%) [5]. Earlier diagnosis greatly increases patient's chances of survival as the mouth is very accessible for a clinical or self-examination [6]. However, oral cancer is still frequently diagnosed in advanced stages. One of the main reasons may be the lack of information about the causes and knowledge of signs and symptoms of oral cancer among the population. Moreover, most of oral cancers are preventable if people know which risk factors they must control or eliminate. In Portugal, a study on oral cancer awareness and knowledge has not been performed.

The purpose of this study was to examine the awareness and knowledge of oral cancer in a hospital population of the city of Valongo in Portugal which could eventually aid in the planning of appropriate health promotion.

## 2. Study Population and Methods

We conducted a cross-sectional study to assess the awareness and knowledge of oral cancer including its risk factors, signs and symptoms, and beliefs in a population attending to the Hospital of Nossa Senhora da Conceição of Valongo (HNSCV), in the north of Portugal. Face-to-face interviews with patients over the age of 18 were conducted by previously trained and calibrated interviewers, between the 10th of January and the 4th of April, 2011. The authorization and ethical approval were obtained from the HNSCV. The consented individuals were interviewed consecutively in the order of their arrival to the hospital, following their consent to the program. Of the 631 subjects invited to participate in the survey, 29 declined or abandoned the inquiry. 

The questionnaire used (available as a web link and on request from the corresponding author) was based on similar previous works [6, 7]. However, we adapted some questions to the Portuguese population. A pilot survey of 30 subjects was conducted and then some questions were modified. 

The inquiry started with a standardized presentation of the interviewer as a student or a volunteer at HNSCV undertaking a study on cancer information (initially without mentioning words like oral cancer, mouth, or dentist). The questionnaire with 26 questions was divided in three parts: (1) a section on sociodemographic aspects; (2) a main questionnaire with questions on oral cancer awareness and knowledge and cancer beliefs; (3) the concluding section with information on lifestyle habits on smoking, alcohol consumption, fruit and vegetable intake, dentist visits, and oral hygiene habits (i.e., toothbrushing frequency). To assess oral cancer awareness, we asked *“Of the following cancers which ones do you know of or have heard of?”* listing a number of cancers including oral cancer. Then we asked *“Have you heard about oral cancer screening? Have you had your mouth examined as a part of oral cancer detection?”*. Response categories for these questions were *“yes*,*” “no,”* or *“don't know”*. Knowledge on oral cancer was assessed by prompted questions relative to oral cancer's frequency in different age groups (0–17, 18–24, 25–44, over 45-year old, do not know), gender (male, female), frequently affected location in mouth (gum, palate, tongue, floor of the mouth, cheek, do not know), world incidence (oral cancer is within the ten most common cancers in the world (yes, no, do not know), oral cancer deaths (<24%, 50%, >75%, do not know), signs and symptoms, and causes by listing a number of items on these topics. We evaluated some individual oral cancer beliefs using three questions: *“Getting oral cancer is a matter of luck and we cannot do anything to prevent this?”*, *“The early discovery of this cancer can increase the success of your treatment”*, *“Do you think we can change our lives to reduce the risk of cancer of the mouth?”*. Response categories for these 3 questions were *“agree,” “disagree,”* or *“don't know.”*

Subjects who smoked cigarettes (or equivalents) at the rate of 20 or more per day were considered heavy smokers. Those who drank more than 14 units of alcohol per week (females) and more than 21 units per week (males) were considered as heavy drinkers [8]. The daily intake of vegetables and fresh fruits were categorized as consuming either five portions or more per day, or not [9]. The questionnaire is available on request from the authors.

### 2.1. Statistics

Each questionnaire was coded and entered into a Microsoft Excel database. To assess the reliability of interview data, completed interviews were subjected to a 10% field check by a process of reinterviews. The results were expressed in absolute and relative frequencies. The relationship between demographic and personal habits versus cancer awareness, cancer knowledge, and cancer beliefs were analyzed by chi-square test. Differences were considered statistically significant at *P <* 0.05.

## 3. Results 

Of the 602 individuals participating in the inquiry 381 (63.3%) were females and 221 (36.7%) were males. The mean age was 48.38 years (SD ± 15.2), in the age range of 18 and 86 years. Five hundred and ninety-nine were White Caucasians (99.5%). Other demographic characteristics of the respondents are showed in Table 1.

Breast cancer was the cancer that most subjects were aware of (99%) followed by lung cancer (96.3%) and prostate cancer (95.2%). Oral cancer was the least heard type of cancer (68.6%) (Table 2). Individuals with better oral hygiene habits were more aware of oral cancer (*n* = 375; 70.5%) than individuals with less frequent oral hygiene (*n* = 38; 54.3%). Also, individuals reporting visits to the dentist more than once a year were more aware of oral cancer (*n* = 195; 74.1%) than less frequent attenders (*n* = 218; 64.3%) (*P* = 0.010). 

Only 120 (19.9%) individuals had heard of oral cancer screening. Ten (1.7%) have had a mouth examination as part of oral cancer screening. 

When asked which was the cancer most easier to detect (by health professionals), 90 (15%) mentioned lung cancer, 339 (56.3%) breast cancer, 15 (2.5%) colon cancer, 42 (7%) oral cancer, and 10 (1.7%) pancreatic cancer. The remaining 106 (17.6%) could not answer this question. 

Only 131 (21.8%) individuals mentioned that oral cancer affected more males than females. Twenty-eight (4.7%) individuals answered that it affected more females, 369 (61.3%) that it affects both sexes equally, and 74 (12.3%) did not answer. 

Most subjects (*n* = 461; 76.6%) did not know that head and neck cancer is within the 10 most frequent cancers in the world. When we asked the age group with the highest incidence of oral cancer, 12 (2%) responded 0–18 years, 28 (4.7%) responded 18–25 years, 146 (24.3%) 25–44 years, and 260 (43.2%) said that it was more frequent in people 45 years or older. 156 (25.9%) individuals reported they do not know the correct answer.

The location of the mouth most referred to as likely to develop oral cancer was the tongue (*n* = 204; 33.9%), followed by gums (*n* = 133; 22.1%), palate (*n* = 61; 10.1%), floor of the mouth (*n* = 29; 4.8%), and cheek mucosa (*n* = 28; 4.7%). Again 147 (24.4%) individuals reported they do not know the correct answer.

Only 86 (14.3%) subjects cited oral cancer mortality to be around 50%. Two hundred and thirty-one individuals (38.4%) referred a mortality lower than 25% and 117 (19.4%) above 75%. The remaining 168 (27.9%) could not respond to this question.

In relation to the causes of oral cancer, 539 (89.5%) individuals identified tobacco and 381 (63.3%) alcohol as a cause of oral cancer. It is curious to note that 194 (32.3%) individuals considered oral cancer was caused by dental treatment at the dental office and another 118 (19.6%) replied it was caused by close contact with other cancer patients (Table 3). 

Patients with smoking habits identified tobacco as a cause of oral cancer (95.7%) more frequently than the nonsmokers (86.9%) (*P* = 0.048). Further significant findings were noted in relation to other demographic factors of the sample and their knowledge on risk factors, but the clinical relevance of these findings is obscure (Table 1).

Ninety percent (*n* = 542) of the individuals were able to identify “an ulcer that does not heal” and 541 (89.9%) a “lump/tissue that overgrows” as signs of oral cancer (Table 4). However, only 318 (52.8%) identified a white or red spot or patch as a sign of oral cancer. The reference that “an ulcer that does not heal” could be a sign of oral cancer was correlated with frequent oral hygiene (*P* = 0.001), and frequency of dental visits (*P* = 0.043). The reference that “lump or tissue that overgrows” could be a sign of oral cancer was correlated with frequent oral hygiene (*P* = 0.012) and higher education level (*P* < 0.001) (Table 1). 

Sixty-one percent (*n* = 364) of the subjects disagreed with the affirmation that *“having oral cancer is a question of luck and there is nothing we can do to avoid it*.*”* However, 169 (28.1%) agreed with this affirmation and 69 (11.5%) could not form an opinion on this. Disagreement was associated with younger age (*P* < 0.001), single marital status (*P* = 0.001), high professional class (*P* = 0.001), high education level (*P <* 0.001), lower alcohol consumption (*P = *0.033), frequent oral hygiene (*P <* 0.001), and frequent dentist visits (*P* < 0.001) (Table 5). 

Most subjects (*n* = 569; 94.5%) agreed that the detection of oral cancer in early stages could increase the success of the treatment. This was rejected by 12 subjects (1.9%), and 21 (3.5%) were unable to respond. Married marital status (*P* = 0.012), history of alcohol consumption (*P* = 0.032), and frequent oral hygiene (*P* < 0.001) were significantly related to agreeing to this answer (Table 5).

 Finally, when we asked the subjects if they agree that *“we can change our lifestyle to reduce the risk of cancer of the mouth*,*”* 495 (82.2%) said yes, 40 (6.6%) disagreed with the affirmation, and 67 (11.1%) could not form an opinion. Positive answers were associated with younger age (*P* < 0.001) and higher education level (*P* = 0.001) (Table 5).

## 4. Discussion

Valongo is a northern Portuguese municipality with 93,573 inhabitants consisting of five local administrative units including Valongo, Ermesinde, Alfena, Sobrado, and Campo. It has one public district hospital that receives all citizens of Valongo. Almost every patient invited to this survey had accepted to participate (95.4%) thus limiting any bias in generalizing our findings to other patients attending this hospital. The predominance of female individuals in the survey (60%) is in accordance with other reports [7] and may be explained by random variation or by the fact that there is a greater proportion of women attending hospitals.

The oral cancer awareness and knowledge in the present population is poor. It is curious to note that oral cancer was the least mentioned of all cancers (68%), even less than pancreatic cancer that has a lower incidence comparing to oral cancer. Our results are in line with the reports by Cruz et al. [10] in USA with an awareness of 66%, lower than that reported by Ariyawardena and Vithanaarachchi [11] (awareness of 95%) and West et al. [12] (awareness of 96%) but a slightly higher than Prayman et al. [7] and Warnakulasuriya et al. [6] with an awareness of 55% and 56%, respectively. These data come from various populations researched over a decade or so.

That smoking is a risk factor for oral cancer was realized by the majority of the subjects. However, alcohol was identified to a lesser degree as a risk factor. This trend was also reported by others [6, 7, 10, 13–15]. It may be related to general health campaigns that primarily promoted tobacco cessation. This groups' awareness on alcohol as a risk factor was lower to the observed figures by Palencia et al. [16]. A recent retrospective study conducted in a head and neck unit in Lisbon, Portugal, reported consumption of alcohol to be the major risk factor for tongue cancer in their cohort of cancer patients [17]. For Portugal, strengthening people knowledge on their increased cancer risks by alcohol use should be included in future health promotion strategies.

Although the presence of a nonhealing wound and oral lumps was widely identified as one of the first signs of oral cancer, the presence of white or red persistent plaques was not associated with oral cancer by almost half of the individuals. This is in accordance with other reports [12, 13]. This can be problematic because in addition to being an early sign of cancer they can correspond to potentially malignant disorders whose elimination in time could contribute to reducing the risk of developing mouth cancer. 

A disheartening 98.5% of the individuals were never submitted to a consultation or had knowledge of been screened for oral cancer. Comparing with other reports this result is one of the lowest published in scientific literature [10, 15, 18]. This is a drawback in our healthcare system that needs to be strengthened in Portugal. Educational campaigns at the population level should be established to transmit accurate information and motivation towards oral cancer prevention. The design of oral cancer screening campaigns properly calibrated becomes urgent, especially among the individuals with high risk for oral cancer. Opportunistic oral cancer screening examination undertaken when patients attend a healthcare professional specially a dentist may be important in this view. However, they must be complemented with other primary care activities because, as we observed in our work, many subjects did not go to the dentist regularly. A network of private and public dentists, and other healthcare professional at health centers or hospitals including GP, nurses, would form an ideal oral cancer screening team providing access for everyone. Primary healthcare professionals can contribute to efficient oral cancer screening [19]. Moreover, this should be complemented with active campaigns of awareness and knowledge on oral cancer by health institutes or the government. Recently, at the end of this study, our service in association with the Valongo municipality administration held an oral cancer screening day for the population of Valongo, an initiative very encouraging and that should serve as an example for other similar activities. 

A recent population-based survey of adults in the UK has shown that a combination of public education of symptoms and empowerment to seek medical advice, as well as support at the primary care level, could enhance early presentation and improve cancer outcomes [20]. A systematic review has reported some evidence that interventions delivered at individual or community level may increase cancer awareness [21]. However, whether awareness leads to early presentation of oral cancer is being debated, and evidence is somewhat limited [22]. Early diagnosis and rapid access for treatment of cancer is an important factor for improving outcomes for oral cancer. Programs such as awareness campaigns and population screenings remain important public health measures to reduce delays in diagnosis.

In the present work, although most of the subjects agree that the detection of oral cancer in early stages can increase the success of the treatment, approximately one-third believed that whether a person developed an oral cancer or not was a matter of luck and therefore was unavoidable. This may be associated to a fatalism concept also observed by Warnakulasuriya et al. [6]. Fatalism attitude to health might be a critical obstacle to changing lifestyles and may be responsible in people either not accepting professional advice on avenues for prevention or arriving too late for the therapy. Associations of this fatalism with old age, poor education and professional class, and heavy alcohol consumption were evident in the present work. Education of the public, particularly young people, may help to change the common attitude that cancer affliction is a matter of chance.

A limitation of our study that we would like to acknowledge is that we surveyed a hospital population attending outpatient clinics. It would have been ideal to survey a random sample of the general population, but our resources were limited. We therefore would caution generalizing the findings of this survey to Portuguese people. 

## 5. Conclusion

This survey highlights the general lack of awareness and knowledge on oral cancer among a population of Valongo. Almost all individuals had never been submitted to oral cancer screening nor had knowledge of been examined for case detection. An oral health promotion strategy should involve elements of basic education on oral cancer as well oral cancer screening that should be implemented for this population.

## Figures and Tables

**Table 1:** Sociodemographic characteristics and their relations with some awareness and knowledge variables.

Variables	*n*	Oral cancer awareness (*n* = 413; 68.6%)	Oral cancer screening (*n* = 120; 19.9%)	Tobacco as risk factor (*n* = 539; 89.5%)	Alcohol as risk factor (*n* = 381; 63.2%)	Ulcer as sign (*n* = 542; 90%)	Red/white patch as sign (*n* = 318; 52.8%)	Swelling as sign (*n* = 541; 89.9%)
Gender								
Male	221	143 (64.7)	48 (21.7)	206 (93.2)	145 (65.6)	193 (87.3)	112 (50.7)	191 (86.4)
Female	381	270 (70.9)	72 (18.9)	333 (87.4)	236 (61.9)	349 (91.6)	206 (54)	350 (91.9)
*P *		0.116	0.404	0.080	0.108	0.225	0.139	0.096
Age								
<49 years	298	198 (66.4)	52 (17.4)	274 (91.9)	186 (62.4)	266 (89.2)	148 (49.7)	269 (90.3)
≥49 years	304	215 (70.7)	68 (22.4)	265 (87.2)	195 (64.1)	276 (90.8)	170 (55.9)	272 (89.5)
*P *		0.258	0.131	0.117	0.275	0.050	0.305	0.518
Professional class								
I/II	56	43 (76.8)	13 (23.2)	52 (92.9)	30 (53.6)	51 (91.1)	35 (62.5)	53 (94.6)
III	51	34 (66.6)	11 (21.6)	47 (92.2)	31 (60.8)	47 (92)	26 (50.9)	48 (94.1)
IV	188	124 (65.9)	30 (15.9)	167 (88.8)	119 (63.3)	166 (88.3)	97 (51.6)	170 (90.4)
V	307	212 (69.1)	66 (21.5)	273 (88.9)	201 (65.4)	278 (90.6)	160 (52.1)	270 (87.9)
*P *		0.481	0.424	0.358	0.385	0.722	0.741	0.375
Marital status								
Single	112	76 (67.8)	20 (17.9)	110 (98.2)	75 (66.9)	100 (89.3)	56 (50)	103 (92)
Married	402	275 (68.4)	77 (19.2)	355 (88.3)	256 (63.7)	361 (89.9)	216 (53.7)	362 (90)
Widow/divorced/separated	88	62 (70.5)	23 (26.1)	74 (84)	50 (56.8)	81 (92)	46 (52.3)	76 (86.4)
*P *		0.916	0.276	**0.014**	0.177	0.290	0.672	0.479
Education								
Without	50	34 (68)	10 (20)	46 (92)	32 (64)	39 (78)	23 (46)	39 (78)
Primary school	429	286 (66.6)	86 (20)	378 (88.1)	279 (65)	386 (89.9)	219 (51)	386 (89.9)
High-school	81	58 (71.6)	15 (18.5)	74 (91.4)	49 (60.5)	77 (54.3)	49 (60.4)	75 (92.6)
Technic-vocation/university	42	35 (83.3)	9 (21.4)	41 (97.6)	21 (50)	40 (95.2)	27 (64.3)	41 (97.8)
*P *		0.149	0.983	0.271	0.083	0.054	0.336	**<0.001**
Smoking								
Nonsmoker	367	258 (70.3)	77 (21)	319 (86.9)	221 (60.2)	330 (90)	203 (55.3)	328 (89.4)
Smoker	141	93 (65.9)	30 (21.3)	135 (95.7)	94 (66.7)	126 (89.4)	61 (43.3)	131 (92.9)
Ex-smoker	94	62 (68)	13 (13.8)	85 (90.4)	66 (70.2)	86 (91.5)	54 (57.4)	82 (87.2)
*P *		0.534	0.272	**0.048**	0.126	0.060	0.088	0.077
Heavy smoking								
No	517	359 (69.4)	107 (20.7)	460 (88.9)	323 (62.5)	464 (89.7)	276 (53.4)	465 (89.3)
Yes	85	54 (63.5)	13 (15.3)	79 (92.9)	58 (68.2)	78 (91.7)	42 (49.4)	76 (89.4)
*P *		0.277	0.248	0.385	0.585	0.359	0.716	0.222
Alcohol								
Nonconsumer	253	170 (67.2)	47 (18.2)	225 (88.9)	157 (62)	226 (89.3)	141 (55.7)	231 (91.3)
Consumer	301	205 (68.1)	72 (23.9)	274 (91)	192 (63.8)	276 (91.7)	153 (50.8)	270 (89.7)
Ex-consumer	48	38 (79.2)	11 (22.9)	40 (83.3)	32 (66)	40 (83.3)	24 (50)	40 (83.3)
*P *		0.252	0.725	0.497	0.121	0.128	0.768	0.502
Heavy alcohol								
No	534	366 (68.5)	102 (19.1)	478 (89.5)	335 (62.7)	479 (89.7)	286 (53.6)	480 (89.9)
yes	68	47 (69.1)	18 (26.5)	61 (89.7)	46 (67.6)	63 (92.6)	32 (47)	61 (89.7)
*P *		0.923	0.152	0.867	0.662	0.716	0.489	0.774
Fruit and vegetables								
<5 units/day	459	322 (70.2)	91 (19.8)	406 (88.5)	283 (61.7)	410 (89.3)	242 (52.7)	412 (89.7)
≥5 units/day	143	91 (63.6)	29 (20.2)	133 (93)	98 (68.5)	132 (92.3)	76 (53.1)	129 (90.2)
*P *		0.143	0.906	0.173	0.235	0.531	0.621	0.892
Oral hygiene frequency								
<1 per day	70	38 (54.3)	15 (21.4)	63 (90)	42 (60)	55 (78.6)	31 (44.2)	56 (80)
≥1 per day	532	375 (70.5)	105 (19.7)	476 (89.4)	339 (63.7)	487 (91.5)	287 (53.1)	485 (91.2)
*P *		**0.006**	0.739	0.957	0.831	**0.001**	0.296	**0.012**
Dentist visits								
<1 year	339	218 (64.3)	60 (17.7)	300 (88.6)	204 (60.2)	299 (88.2)	172 (50.7)	299 (88.2)
≥1 per year	263	195 (74.1)	60 (22.8)	239 (90.9)	177 (67.3)	243 (92.4)	146 (55.5)	242 (92)
*P *		**0.019**	0.119	0.465	0.149	**0.043**	0.231	0.234

**Table 2:** Most mentioned cancers in the survey.

Type of cancer	(*n*)	(%)
Breast	596	99
Lung	580	96.3
Prostate	574	95.2
Stomach	546	90.7
Colon	532	88.4
Pancreas	519	86.2
Thyroid	482	80.1
Cervical	477	79.2
Skin	474	78.7
Mouth	413	68.6

**Table 3:** Most risk factors or causes mentioned for oral cancer.

Causes of cancer	(*n*)	(%)
Tobacco	539	89.5
Reduced oral hygiene	501	83.2
Infections in the teeth	492	81.7
Alcohol	381	63.3
Reduced intake fruit and vegetables	295	49
Sun exposure	294	48.8
Car smoke	283	47
Dental treatment in dentists	194	32.2
Coffee	137	22.8
Close contact with another cancer patient	118	19.6

**Table 4:** Mostly mentioned early manifestations of oral cancer.

Signs and symptoms	*n*	%
Sore (ulcer) that does not heal	542	90
Lump or tissue overgrow	541	89.9
Difficulty in swallowing	390	64.8
Hemorrhage	370	61.5
Abscess, boil, or infection	359	59.6
Difficulty in open the mouth	338	56.1
Persistent white or red patch	318	52.8
Gastric pain	250	41.5
Prosthesis that fails to fit	201	33.4
Having aphthae very often	163	27.1

**Table 5:** Socio-demographic characteristics and their relations with some cancer beliefs variables.

	*n*	Disagree with question of luck (*n* = 364; 60.5%)	Early detection can improve treatment (*n* = 569; 94.5%)	Lifestyle influence risk of oral cancer (*n* = 495; 82.2%)
Gender				
Male	221	134 (60.6)	211 (100)	187 (84.6)
Female	381	230 (60.4)	358 (94)	308 (80.8)
*P *		0.949	0.432	0.243

Age				
<49 years	298	216 (72.5)	284 (95.3)	262 (87.9)
>49 years	304	148 (48.7)	285 (93.7)	233 (76.6)
*P *		**<0.001**	0.403	**<0.001**

Professional class				
I/II	56	46 (82.1)	53 (94.6)	50 (89.2)
III	51	34 (66.6)	49 (96)	45 (88.2)
IV	188	115 (61.1)	175 (93)	157 (83.5)
V	307	169 (55)	292 (95.1)	243 (79.1)
*P *		**0.001**	0.754	0.147

Marital status				
Single	112	85 (75.8)	110 (98.2)	99 (88.4)
Married	402	233 (57.9)	381 (94.8)	328 (81.6)
Widow/divorced/separated	88	46 (52.3)	78 (88.6)	68 (77.3)
*P *		**0.001**	**0.012**	0.105

Education				
Without	50	21 (42)	45 (90)	34 (68)
Primary school	429	236 (55)	404 (94.1)	348 (81.1)
Secondary	81	69 (85.2)	79 (97.5)	73 (90.1)
Technic-vocation/university	42	38 (90.5)	41 (97.6)	40 (95.2)
*P *		**<0.001**	0.234	**0.001**

Smoking				
Nonsmoker	367	214 (58.3)	345 (94)	297 (80.9)
Smoker	141	97 (68.8)	135 (95.7)	123 (87.2)
Ex-smoker	94	53 (56.4)	89 (94.7)	75 (79.8)
*P *		0.065	0.741	0.199

Heavy smoking				
No	517	313 (60.5)	489 (94.6)	426 (82.4)
Yes	85	51 (60)	80 (94.1)	69 (81.1)
*P *		0.925	0.861	0.761

Alcohol				
Nonconsumer	253	157 (62)	232 (91.7)	200 (79)
Consumer	301	186 (61.8)	290 (96.3)	255 (84.7)
Ex-consumer	48	21 (43.8)	47 (97.9)	40 (83.3)
*P *		**0.047**	**0.032**	0.216

Heavy alcohol				
No	534	331 (61.9)	503 (94.1)	436 (81.6)
yes	68	33 (48.5)	66 (97)	59 (86.8)
*P *		**0.033**	0.328	0.299

Fruit and vegetables				
<5 units/day	459	281 (61.2)	431 (93.9)	375 (81.7)
>5 units/day	143	83 (58)	138 (96.5)	120 (83.9)
*P *		0.497	0.232	0.545

Oral hygiene frequency				
<1 day	70	25 (35.7)	57 (81.4)	56 (80)
1 or more per day	532	339 (63.7)	512 (96.2)	439 (82.5)
*P *		**<0.001**	**<0.001**	0.604

Dentist visits				
<1 year	339	181 (53.3)	315 (92.9)	277 (81.7)
1 or more per year	263	183 (69.6)	254 (96.6)	218 (82.9)
*P *		**<0.001**	0.051	0.707
